# Differential Gene Expression and Withanolides Biosynthesis During *in vitro* and *ex vitro* Growth of *Withania somnifera* (L.) Dunal

**DOI:** 10.3389/fpls.2022.917770

**Published:** 2022-06-14

**Authors:** Sachin Ashok Thorat, Arya Kaniyassery, Poornima Poojari, Melissa Rangel, Shashikala Tantry, Kodsara Ramachandra Kiran, Manjunath B. Joshi, Padmalatha S. Rai, Anna-Maria Botha, Annamalai Muthusamy

**Affiliations:** ^1^Department of Plant Sciences, Manipal School of Life Sciences, Manipal Academy of Higher Education (MAHE), Manipal, India; ^2^Manipal School of Life Sciences, Manipal Academy of Higher Education (MAHE), Manipal, India; ^3^Department of Ageing Research, Manipal School of Life Sciences, Manipal Academy of Higher Education (MAHE), Manipal, India; ^4^Department of Biotechnology, Manipal School of Life Sciences, Manipal Academy of Higher Education (MAHE), Manipal, India; ^5^Department of Genetics, Faculty of Agriculture, University of Stellenbosch, Stellenbosch, South Africa

**Keywords:** adventitious roots, differential gene expression, *in vitro*, *ex vitro*, metabolites, organogenesis, *Withania somnifera*, withanolide

## Abstract

Ashwagandha (*Withania somnifera* L. Dunal) is a medicinally important plant with withanolides as its major bioactive compounds, abundant in the roots and leaves. We examined the influence of plant growth regulators (PGRs) on direct organogenesis, adventitious root development, withanolide biosynthetic pathway gene expression, withanolide contents, and metabolites during vegetative and reproductive growth phases under *in vitro* and *ex vitro* conditions. The highest shooting responses were observed with 6-benzylaminopurine (BAP) (2.0 mg L^–1^) + Kinetin (KIN) (1.5 mg L^–1^) supplementation. Furthermore, BAP (2.0 mg L^–1^) + KIN (1.5 mg L^–1^) + gibberellic acid (GA_3_) (0.5 mg L^–1^) exhibited better elongation responses with *in vitro* flowering. Half-strength MS medium with indole-3-butyric acid (IBA) (1.5 mg L^–1^) exhibited the highest rooting responses and IBA (1.0 mg L^–1^) with highest fruits, and overall biomass. Higher contents of withaferin A (WFA) [∼8.2 mg g^–1^ dry weight (DW)] were detected in the reproductive phase, whereas substantially lower WFA contents (∼1.10 mg g^–1^ DW) were detected in the vegetative phase. Cycloartenol synthase (*CAS*) (*P* = 0.0025), sterol methyltransferase (*SMT*) (*P* = 0.0059), and 1-deoxy-D-xylulose-5-phosphate reductase (*DXR*) (*P* = 0.0375) genes resulted in a significant fold change in expression during the reproductive phase. The liquid chromatography-mass spectrometry (LC-MS) analysis revealed metabolites that were common (177) and distinct in reproductive (218) and vegetative (167) phases. Adventitious roots cultured using varying concentrations of indole-3-acetic acid (IAA) (0.5 mg L^–1^) + IBA (1.0 mg L^–1^) + GA_3_ (0.2 mg L^–1^) exhibited the highest biomass, and IAA (0.5 mg L^–1^) + IBA (1.0 mg L^–1^) exhibited the highest withanolides content. Overall, our findings demonstrate the peculiarity of withanolide biosynthesis during distinct growth phases, which is relevant for the large-scale production of withanolides.

## Introduction

*Withania somnifera* (L.) Dunal, widely known as Ashwagandha, Indian Ginseng, or Winter Cherry, belongs to the Solanaceae family. It is an essential aromatic medicinal plant regularly used in traditional medicine (Singh et al., 2015). The plant possesses multipotential therapeutic properties, and in India, it is used in various Ayurvedic formulations (Shasmita et al., 2018). Studies have shown that the plant is effective for the treatment of various ailments and diseases, such as cancer, asthma, aging, and neurological and inflammatory disorders, due to its anti-cancer, anti-stress, anti-inflammatory, antianxiety, anticonvulsant, adaptogenic, immunomodulatory, endocrine, and cardiovascular activities (Kulkarni and Dhir, 2008; Dar et al., 2015; Singh et al., 2015; Gurav et al., 2020). Its multiple pharmaceutical properties are due to secondary metabolites known as withanolides, withaferin A (WFA), withanolide A (WA), withanolide D (WD), and withanone (WN) (Chatterjee et al., 2010). Recently, this plant has been reported for its potential effectiveness in treating and managing COVID-19 symptoms (Chikhale et al., 2021; Saggam et al., 2021). In addition, *in silico* studies on WN and withanoside V have shown a possible antiviral role in inhibiting the activity of a protease (M^pro^) from SARS-CoV-2 (Kumar et al., 2020).

Withanolides are comprised of thirty carbon compounds known as triterpenoids synthesized from isoprene units through a withanolide biosynthetic pathway. Furthermore, it has divided into two classical pathways, namely, cytosolic mevalonic acid (MVA) and plastidial methylerythritol phosphate (MEP) pathways. Important genes involved in this pathway, such as 3-hydroxy-3-methylglutaryl-coenzyme A reductase (*HMGR*), convert hydroxymethylglutaryl coenzyme A into mevalonate, marking the first step of MVA pathway in the cytosol. In contrast, 1-deoxy-D-xylulose-5-phosphate reductase (*DXR*) gene converts 1-deoxy-d-xylulose-5-phosphate into 2-*o*-methyl-d-erythritol-4-phosphate, marking the first step of the MEP pathway in the plastid. Cycloartenol synthase (*CAS*) and sterol methyltransferase (*SMT*) genes are intermediates in synthesizing 24-methylenecycloartenol and subsequently forming various withanolides (Dhar et al., 2015).

Due to extensive uses in medicinal formulations, the demand for dried plant material for withanolide production has increased worldwide (Sathiyabama and Parthasarathy, 2018). Withanolide biosynthesis is a plant tissue-specific process and depends on the growing conditions in the natural habitat. The traditional cultivation of *W. somnifera* in field conditions is laborious and time-consuming, and the product yield is also affected by a range of biotic and abiotic factors. Thus, these factors influence the biosynthesis of withanolides and their purity, further complicates the analysis of phytochemical composition. This results in an inadequate yield of products that cannot meet the demands in the current international market (Sangwan et al., 2004; Sivanandhan et al., 2012). In contrast, plant biotechnological tools, such as *in vitro* culture propagation, show higher growth rates under optimal growth conditions. This facilitates fast cell proliferation, leading to a significant growth irrespective of seasonal variations. Collectively, *in vitro* culture methods assist in producing callus and plantlets in a controlled environment and can be upscaled to large-scale bioreactors without interferences from biotic and abiotic factors (Rao and Ravishankar, 2002; Verpoorte et al., 2002).

Currently, biotechnological tools are widely used for the conservation, *in vivo* and *in vitro* breeding, selection, and genetic and metabolic engineering of *W. somnifera* genotypes. Plant tissue culture has also shown a great potential for the rapid production of phytomedicines (Singh et al., 2016). Several conventional and molecular experiments have been performed in *W. somnifera* for crop improvement, but none of them have used novel bioactive molecules to enhance growth and metabolite production in *W. somnifera* (Poornima et al., 2019; Sharma et al., 2020; Srivastava and Sangwan, 2020; Thorat et al., 2021). Although several studies are available on the *in vitro* regeneration and the quantification of withanolides, withanolide biosynthetic pathway gene expression, distribution of metabolites during the vegetative and reproductive phases, and adventitious root formation have not been reported. Therefore, this study has evaluated the influence of different combinations of plant growth regulators (PGRs) on multiple shoot initiation, direct organogenesis, plantlet regeneration, *in vitro* and *ex vitro* flowering, and adventitious root formation. Furthermore, we have also looked at withanolide contents, metabolite profile, and differential gene expression of the withanolide biosynthetic pathway during the vegetative and reproductive phases.

## Materials and Methods

### *In vitro* Seed Germination, Multiple Shoot Induction, and Regeneration

The seeds of Ashwagandha var. Jawahar Asgandh-20 were procured from ICAR-Directorate of Medicinal and Aromatic Plants Research, Anand, Gujarat. The seeds were washed under running water for 30 min, treated with 2% Tween 20 for 15 min, and rinsed with distilled water. Then, surface decontamination was achieved using aqueous 0.1% mercuric chloride (w/v), and later seeds were rinsed five times with autoclaved distilled water. The seeds were inoculated aseptically onto half-strength MS (Murashige and Skoog, 1962) and fortified with 0.25 mg L^–1^ gibberellic acid (GA_3_). Then, the seeds were incubated in the dark for 48 h and then exposed to 40 μmol m^–2^ s^–1^ cool-white, fluorescent light with a 16-h photoperiod for germination. The shoot tips, as well as nodal segments, were taken from 3-week-old *in vitro* grown seedlings and inoculated onto MS media with different concentrations of 6-benzylaminopurine (BAP) (0.5, 1.0, 1.5, 2.0, 2.5, and 3.0 mg L^–1^) and Kinetin (KIN) (0.5, 1.0, 1.5, 2.0, 2.5, and 3.0 mg L^–1^) individually, and later well responded to KIN (1.5 mg L^–1^) + BAP (0.5–3.0 mg L^–1^) in various combinations. However, MS without BAP and KIN was eliminated because several studies have demonstrated that it is ineffective on multiple shoot induction and regeneration. After 20 days, the number of shoots >2 cm were counted and noted. The growing shoots were subcultured on the same medium supplemented with GA_3_ for multiplication and elongation. After 40 days, shoots with a minimum height of 2.5 cm were transplanted onto half-strength MS media fortified with different indole-3-butyric acid (IBA) concentrations (0.0, 0.5, 1.0, 1.5, and 2.0 mg L^–1^). The well-rooted plantlets were hardened on a sterile mix of soil, coir, and sand in a 1:1:1 ratio and maintained in the plant tissue culture room for 10 days. Furthermore, the plantlets were transferred to plastic pots in the same combination of soil without sterilization in the greenhouse for acclimatization.

### *In vitro* and *ex vitro* Flowering and Fruit Set

During the induction and development of shoots and roots, the interesting phase of *in vitro* flowering was also observed and noted. Plantlets showed continuously developing flowers *ex vitro* and set fruits. All the *in vitro* and *ex vitro* flowers and fruits were carefully counted from all the treatment groups. In addition, the seeds were collected during *in vitro* and *ex vitro* flowering and assessed for their viability and germination patterns.

### Adventitious Root Culture

Leaf segments (5 mm × 5 mm) along the midrib were excised from 35-day-old *in vitro* seedlings and inoculated onto MS media. The initiation of adventitious roots was observed at the leaf midrib after 30 days of culture. Root segments (around 1.0 cm long), including the root tip, were inoculated on liquid MS media for proliferation. MS media with 0.5 mg L^–1^ indole-3-acetic acid (IAA) in a combination with different IBA (1.0 and 2.0 mg L^–1^) concentrations and without PGRs were used for liquid media. Furthermore, these two combinations were analyzed with and without 0.20 mg L^–1^ GA_3_. The adventitious roots were inoculated in 25 ml liquid MS media and incubated for a 16-h light and 8-h dark photoperiod for 1 week, followed by complete darkness, in an incubation shaker at room temperature (25 ± 2°C) at 80 rpm. The root cultures were maintained in three replicates in conical flasks for each combination of PGRs.

### Expression of Genes Involved in the Withanolide Biosynthetic Pathway

The expression levels of withanolide biosynthetic pathway genes, such as *CAS*, *DXR*, 3-hydroxy-3-methylglutaryl-coenzyme A reductase (*HMGR*), and *SMT*, were examined for a comparative analysis of vegetative and reproductive phase plants. Briefly, 100 mg of leaf tissue from vegetative and reproductive phase plants was powdered using liquid nitrogen, and total RNA was extracted using TRI Reagent^®^ (MRC #TR118) according to the manufacturer’s instructions. Furthermore, the concentration and purity of the RNA were quantified using Nanodrop ND-100 Spectrophotometer (Thermo Scientific, MA, United States). Then, isolated RNAs (2 μg) were treated with DNase I to eliminate DNA contamination using DNa-free™ Kit (Invitrogen™ #AM1906). Later, cDNA was synthesized using a High-Capacity cDNA Reverse Transcription Kit (Applied Biosystems™ #4368814) as directed by the manufacturer’s protocol. The quantitative real-time expression of genes was performed with QuantStudio 6 Pro Real-Time PCR systems (Applied Biosystems™, United States) using PowerUp™ SYBR™ Green Master Mix (Applied Biosystems™ #A25741).

The abundance of transcripts in the samples was determined by the relative quantification of mRNA using the comparative cycle threshold (*Ct*) method. Furthermore, the *ACTIN* gene was used as constitutively expressed control to normalize the quantity of template cDNA. The quantitative relative expression of mRNA was determined by the 2^–ΔΔCT^ method (Kiran et al., 2020). The primers’ list and their sequences are depicted in Supplementary Table 1.

### Extraction and Quantitative Analysis of Withanolides

The hardened plantlets were used for the extraction of withanolides. The plantlets and adventitious roots were harvested, washed thoroughly, and dried at 40°C in a hot air oven for 3 days, and 1 g of plant materials (roots, shoots, and leaves) was grounded into a fine powder with the help of a mortar and pestle. Analytical grades, namely WFA, WA, withanolide B (WB), and WN, were purchased from Natural Remedies Pvt Ltd. (Bangalore, India) and used as standards. The fine powder was extracted thrice with 25% methanol in a shaker incubator at 37°C and filtered with Whatman filter paper at every 2 h interval, followed by depigmentation and defatting with an equal volume of n-hexane three times; finally, the methanol: water phase was separated and defatted with an equal volume of chloroform for three times; chloroform phase was separated and evaporated till dryness at 40°C in a vacuum concentrator (Eppendorf^®^) and stored at −20°C till HPLC analysis (Sangwan et al., 2008).

The samples were re-dissolved in a known volume of methanol and filtered through a 0.2-μm syringe filter (Minisart, Sartorius Stedim Biotech) for HPLC analysis. The withanolides were quantitatively analyzed using Waters Alliance HPLC 2695 with Separation Module System consisting of Dual λ Absorbance Detector 2487 and Phenomenex Luna-C18 column (250 mm × 4.6 mm) and monitored using the Empower 2Pro software. A binary gradient of water (solvent A) and methanol (solvent B), both containing 0.1% formic acid (FA), was used. Gradient system was carried out at 25°C and was initially at 60 A/40 B and gradually changed to 40 A/60 B in 12.0 min duration, maintained for the next 2.0 min, then changed to 25 A/75 B at 14 min, and then to 5 A/95 B at 16.0 min at a flow rate of 1.0 ml min^–1^. The compounds were detected, and peaks were assigned by running the four commercial withanolide standards of known concentration and consequent comparison of retention times. The calibration curve was generated with respective standards. The WFA, WA, WB, and WN contents were identified by comparing retention time with standards, and the concentration was calculated using a regression equation obtained from a calibration curve using the Empower 2 pro software.

### Liquid Chromatography–Mass Spectrometry Analysis

Whole plant samples were dried in a hot air oven at 40°C for 3 days. Later, 100 mg of each representative sample (whole plant) from vegetative and reproductive phases was pooled and grounded to a fine powder using liquid nitrogen in a pre-chilled mortar and pestle. Total metabolites were extracted using 1 ml of chilled acidified methanol comprising 99.875% of methanol and 0.125% of FA. Then, the extract was passed through a 0.2-μm syringe filter (Minisart, Sartorius Stedim Biotech) and stored at −20°C. Furthermore, the extract was filtered with a 0.2-μm syringe filter (Minisart, Sartorius Stedim Biotech) and stored at −20°C. The untargeted metabolite profiling of *W. somnifera* was achieved using Agilent 6530 Accurate-Mass Q-TOF liquid chromatography-mass spectrometry (LC-MS) HPLC ESI system (Agilent Technologies, Santa Clara, CA, United States). The metabolites separation was performed using a 250 mm × 4.6 mm C18 column (Phenomenex-Luna) with mobile phases consisting of solvent A (0.1% FA in Milli-Q water) and solvent B (0.1% FA in acetonitrile). The injection volume of 8 μl was used with a 0.5-ml min^–1^ flow rate and 70 min run time through positive ESI mode in triplicates through gradient method and with maintained ion range (Kiran et al., 2021; Swathy et al., 2021). Metabolites were identified using the Plantcyc online database based on their *m/z* values, and each compound was represented by its molecular formula, retention time, and mass error (≤ ± 30 ppm).

### Statistical Analysis

All the tissue culture experiments were conducted in triplicates with 10 explants in each group, and values in the table were expressed as mean ± standard error (SE). The cultures were subcultured every 15 days, observed periodically for the percentage of responses in each stage, and noted. The values of the experiments were subjected to statistical analysis with analysis of variance (ANOVA) and compared using Duncan’s multiple range tests (Gomez and Gomez, 1984). The gene expression significance values were analyzed using multiple *t*-test, and all the graphical figures were made using the GraphPad 8 Prism software.

## Results

### Multiple Shoot Initiation From Shoot Tip Explants

**FIGURE 1:**
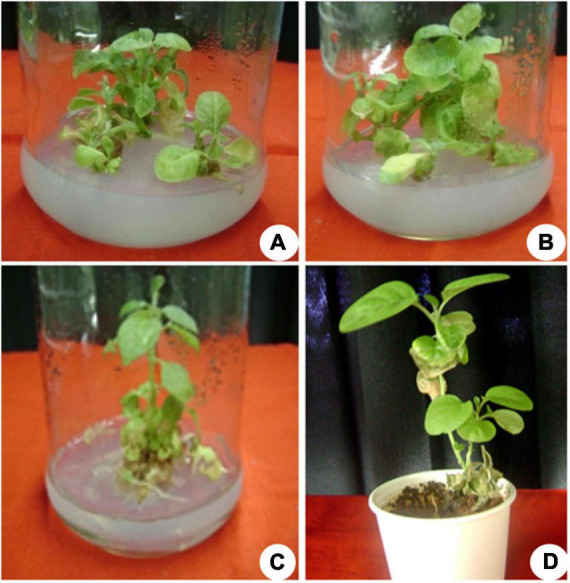
The *in vitro* culture of *Withania somnifera*. **(A)** Shoot multiplication (scale = 2.5 cm), **(B)** shoot elongation (scale = 3.6 cm), **(C)** shoot elongation and rooting (scale = 5.8 cm), and **(D)** hardening of plantlets (scale = 7.7 cm).

Shoot tip explants inoculated on MS basal medium supplemented with different PGRs, either alone or in combination with varying concentrations of BAP and KIN (0.5–3.0 mg L^–1^), had shown a differential response in multiple shoot initiation, shoot length, and days required for shoot initiation (Table 1). The maximum number of shoots (9.0) was noted on the BAP (2.0 mg L^–1^) + KIN (1.5 mg L^–1^), followed by BAP (1.0 mg L^–1^) + KIN (1.5 mg L^–1^) and BAP (1.5 mg L^–1^) + KIN (1.5 mg L^–1^), and the lowest was noted on BAP (3.0 mg L^–1^). The minimum number of days (30.0) required for shoot initiation was noted on BAP (2.0 mg L^–1^) and KIN (1.5 mg L^–1^) followed by BAP (2.0 mg L^–1^) + KIN (1.5 mg L^–1^), whereas the delayed (42.0 days) shoot initiation was noted on BAP (3.0 mg L^–1^) + KIN (1.5 mg L^–1^). The maximum shoot length (5.36 ± 0.40 cm) was noted in BAP (2.0 mg L^–1^) + KIN (1.5 mg L^–1^), followed by KIN (2.0 mg L^–1^) and BAP (1.0 mg L^–1^) + KIN (1.5 mg L^–1^) (Figures 1A–D).

**TABLE 1:** *In vitro* responses of shoot tip explants of *Withania somnifera* to different concentrations of PGRs.

Concentrations of PGRs (mgL^–1^)	Number of shoots/explant	Days to initiation of multiple shoots	Length of shoots (cm)
BAP			
0.5	3.0 ± 0.57b	37.0 ± 3.51ab	2.60 ± 0.10e
1.0	3.0 ± 0.57b	34.0 ± 2.64c	2.93 ± 0.30d
1.5	4.0 ± 0.57a	33.0 ± 2.51cd	3.50 ± 0.30b
2.0	4.0 ± 1.15a	30.0 ± 1.52e	3.86 ± 0.30a
2.5	3.0 ± 1.0b	34.0 ± 2.64c	3.40 ± 0.10bc
3.0	2.0 ± 0.57c	38.0 ± 2.51ab	3.20 ± 0.20c
**KIN**			
0.5	4.0 ± 1.0b	34.0 ± 2.51c	3.70 ± 0.30d
1.0	5.0 ± 0.57a	39.0 ± 1.52a	4.0 ± 0.20c
1.5	5.0 ± 1.0a	30.0 ± 1.52e	4.26 ± 0.25b
2.0	4.0 ± 1.15b	33.0 ± 3.0cd	4.76 ± 0.25a
2.5	4.0 ± 0.57b	37.0 ± 3.51b	4.0 ± 0.20c
3.0	3.0 ± 0.57c	37.0 ± 3.51b	3.86 ± 0.20cd
**BAP and KIN**			
0.5 + 1.5	5.0 ± 0.57d	37.0 ± 1.52bc	4.03 ± 0.15de
1.0 + 1.5	7.0 ± 0.57b	38.0 ± 3.05b	4.43 ± 0.20b
1.5 + 1.5	7.0 ± 0.57b	37.0 ± 2.0bc	4.4 ± 0.26bc
2.0 + 1.5	9.0 ± 0.57a	32.0 ± 2.0d	5.36 ± 0.40a
2.5 + 1.5	6.0 ± 0.57c	38.0 ± 2.64b	4.06 ± 0.20d
3.0 + 1.5	5.0 ± 0.57d	42.0 ± 1.52a	3.83 ± 0.15e

*Mean values within a column having same alphabet are not significantly different (p < 0.05) according to Duncan’s multiple range test (DMRT). Data are the mean and standard deviation for n = 3 independent experiments.*

### Multiple Shoot Initiation From Nodal Explants

**FIGURE 2:**
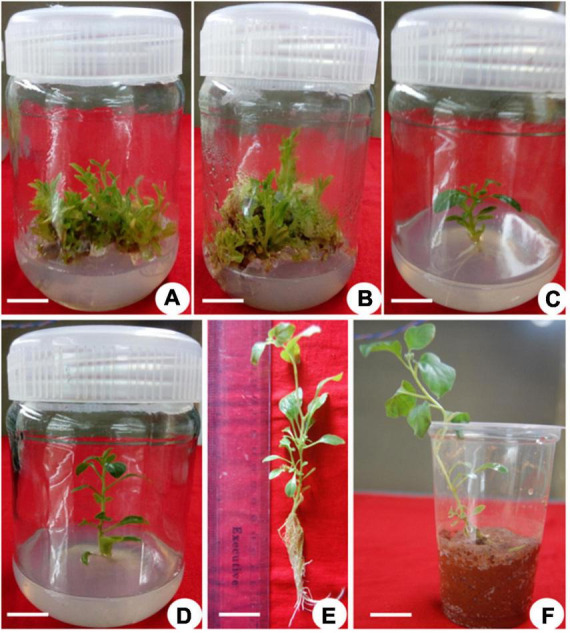
Multiple shoot induction in *Withania somnifera*. **(A)** Induction of multiple shoots from nodal explant with 6-benzylaminopurine (BAP) and Kinetin (KIN) (scale bar = 0.5–1.3 cm), **(B)** multiplication of shoots with BAP and TDZ (scale bar = 1.00–3.05 cm), **(C)** elongation of shoots using BAP and KIN (scale bar = 2–5.3 cm), **(D)** induction of roots from elongated shoots [indole-3-butyric acid (IBA) 0.3 mg/L] (scale bar = 6.5 cm), **(E)** well-developed plantlets (scale bar = 18 cm), and **(F)** hardening of plantlets at 25 ± 2 °C.

Nodal explants were inoculated on MS basal medium supplemented with different PGRs, either alone or in combination with varying concentrations of BAP and KIN (0.5–3.0 mg L^–1^), showing multiple shoot initiations with shoot lengths and times required for shoot initiation at varied frequencies (Table 2). The maximum number of shoots (23.0) was noted with BAP (2.0 mg L^–1^) + KIN (2.0 mg L^–1^), followed by BAP (2.0 mg L^–1^), BAP (2.5 mg L^–1^), and BAP (2.5 mg L^–1^) + KIN (1.5 mg L^–1^), and the lowest was noted with KIN (0.5 mg L^–1^). Among different PGRs, early shoot initiation (28.0 days) was noted on MS fortified with BAP (2.0 mg L^–1^), followed by BAP (2.0 mg L^–1^) + KIN (1.5 mg L^–1^) and KIN (2.0 mg L^–1^), whereas the delayed (42.0 days) shoot initiation was noted with BAP (3.0 mg L^–1^) + KIN (1.5 mg L^–1^). The maximum shoot length (6.03 cm) was noted in BAP (2.0 mg L^–1^), followed by BAP (2.0 mg L^–1^) + KIN (1.5 mg L^–1^) (Table 2 and Figures 2A,B).

**TABLE 2:** *In vitro* responses of nodal explants of *Withania somnifera* to different concentrations of PGRs.

Concentrations of PGRs (mgL^–1^)	Number of shoots per explant	Days for initiation of multiple shoots	Length of the shoots (cm)
BAP			
0.5	10.0 ± 1.15d	34.0 ± 2.64bc	3.03 ± 0.20de
1.0	10.0 ± 1.15d	33.0 ± 2.51c	3.56 ± 0.32d
1.5	13.0 ± 1.0c	32.0 ± 1.15cd	4.13 ± 0.25c
2.0	18.0 ± 2.51a	28.0 ± 1.52e	6.03 ± 0.20a
2.5	17.0 ± 1.52ab	36.0 ± 2.08b	5.13 ± 0.35b
3.0	10.0 ± 2.0d	40.0 ± 2.08a	4.06 ± 0.3cd
**KIN**			
0.5	8.0 ± 1.52de	38.0 ± 3.05ab	4.13 ± 0.30de
1.0	12.0 ± 1.15c	38.0 ± 1.15ab	4.40 ± 0.30d
1.5	14.0 ± 2.0b	36.0 ± 2.08b	4.96 ± 0.20b
2.0	16.0 ± 2.0a	30.0 ± 3.05c	5.56 ± 0.25a
2.5	12.0 ± 1.52c	36.0 ± 2.64b	4.73 ± 0.11bc
3.0	9.0 ± 2.08d	40.0 ± 1.52a	3.96 ± 0.15e
**BAP and KIN**			
0.5 + 1.5	11.0 ± 20d	39.0 ± 2.08b	4.66 ± 0.15bc
1.0 + 1.5	14.0 ± 1.52c	38.0 ± 1.52bc	4.96 ± 0.15b
1.5 + 1.5	15.0 ± 1.52bc	35.0 ± 3.21cd	4.96 ± 0.20b
2.0 + 1.5	23.0 ± 2.51a	30.0 ± 1.15e	5.80 ± 0.30a
2.5 + 1.5	17.0 ± 1.52b	37.0 ± 2.08c	4.43 ± 0.25c
3.0 + 1.5	14.0 ± 1.52c	42.0 ± 1.52a	4.23 ± 0.11cd

*Mean values within a column having same alphabet are not significantly different (p < 0.05) according to Duncan’s multiple range test (DMRT). Data are the mean and standard deviation for n = 3 independent experiments.*

### Multiplication and Elongation of Shoots and *in vitro* Flowering

**FIGURE 3:**
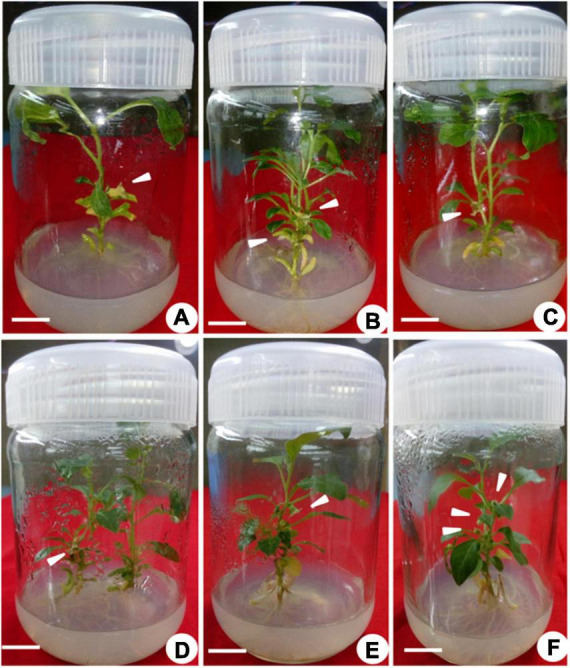
*In vitro* flowering of *Withania somnifera*. **(A)** *In vitro* flower formation from second node (scale bar = 0.7 cm), **(B)** first and second nodes (scale bar = 0.6 and 0.5 cm); **(C,D)** second node (scale bar = 0.6 and 0.4 cm), **(E)** sixth node (scale bar = 0.5 cm), and **(F)** third, fourth, and fifth nodes (scale bar = 0.4, 0.3, and 0.7 cm).

Multiple shoots from the shoot tip and nodal explants were transferred to MS medium supplemented with different PGRs + GA_3_ or in combination. They displayed the effects on elongation, shoot length, number of leaves, and flowers at a varied frequency (Table 3). The highest percentage of shoot elongation (82.0%) was noted on the BAP (2.0 mg L^–1^) + KIN (1.5 mg L^–1^) + GA_3_ (0.5 mg L^–1^), followed by BAP (1.5 mg L^–1^) + KIN (1.5 mg L^–1^) + GA_3_ (0.5 mg L^–1^) and KIN (2.0 mg L^–1^) + GA_3_ (0.5 mg L^–1^) (Figures 1B, 2C). Maximum shoot length (6.56 cm) with a maximum number of leaves (12.0) was noted on BAP (2.0 mg L^–1^) + KIN (1.5 mg L^–1^) + GA_3_ (0.5 mg L^–1^), followed by KIN (2.0 mg L^–1^) + GA_3_ (0.5 mg L^–1^) and BAP (2.0 mg L^–1^) + GA_3_ (0.5 mg L^–1^). A maximum number of flowers *in vitro* (7.0) was noted on BAP (2.0 mg L^–1^) + KIN (1.5 mg L^–1^) + GA_3_ (0.5 mg L^–1^) and BAP (2.0 mg L^–1^) + GA_3_ (0.5 mg L^–1^), respectively, followed by BAP (1.5 mg L^–1^) + KIN (1.5 mg L^–1^) + GA_3_ (0.5 mg L^–1^) and BAP (1.5 mg L^–1^) + GA_3_ (0.5 mg L^–1^) (Table 3 and Figures 3A–F).

**TABLE 3:** Effects of BAP, KIN, and GA_3_ on percentage of elongation, length of shoots, and number of leaves and flowers in *Withania somnifera*.

PGRs (mgL^–1^)	Percentage of elongation	Length of shoots (cm)	Number of leaves	Number of flowers
BAP + GA_3_				
0.5 + 0.5	59.66 ± 4.50e	4.0 ± 0.20de	6.0 ± 1.15c	3.0 ± 0.57e
1.0 + 0.5	64.33 ± 3.21cd	5.56 ± 0.32b	7.0 ± 1.15b	5.0 ± 0.57c
1.5 + 0.5	70.0 ± 2.0b	5.23 ± 0.15bc	7.0 ± 0.57b	6.0 ± 0.57b
2.0 + 0.5	73.0 ± 3.0a	6.36 ± 0.37a	10.0 ± 1.52a	7.0 ± 1.15a
2.5 + 0.5	65.33 ± 1.52c	5.13 ± 0.65c	7.0 ± 0.57b	4.0 ± 1.15d
3.0 + 0.5	59.66 ± 1.52e	4.36 ± 0.35d	6.0 ± 0.57c	3.0 ± 0.57e
**KIN + GA_3_**				
0.5 + 0.5	61.0 ± 3.60d	4.46 ± 0.30de	6.0 ± 0.57d	2.0 ± 0.57d
1.0 + 0.5	66.66 ± 3.05c	5.06 ± 0.85c	7.0 ± 0.57c	4.0 ± 1.52b
1.5 + 0.5	74.33 ± 2.08ab	5.63 ± 0.37b	7.0 ± 0.57c	4.0 ± 0.57b
2.0 + 0.5	76.33 ± 2.08a	6.46 ± 0.15a	10.0 ± 1.15a	5.0 ± 0.57a
2.5 + 0.5	64.0 ± 4.0cd	5.36 ± 0.58bc	8.0 ± 1.52b	4.0 ± 0.57b
3.0 + 0.5	61.66 ± 3.51d	4.63 ± 0.47d	6.0 ± 0.57d	3.0 ± 0.57c
**BAP + KIN + GA_3_**				
0.5 + 1.5 + 0.5	70.33 ± 2.51cd	5.06 ± 0.25de	7.0 ± 0.57c	4.0 ± 0.57d
1.0 + 1.5 + 0.5	73.33 ± 3.05c	5.60 ± 0.20c	8.0 ± 1.15bc	5.0 ± 1.15c
1.5 + 1.5 + 0.5	77.33 ± 2.08b	5.96 ± 0.20b	9.0 ± 0.57b	6.0 ± 1.52b
2.0 + 1.5 + 0.5	82.0 ± 2.64a	6.56 ± 0.20a	12.0 ± 1.15a	7.0 ± 1.52a
2.5 + 1.5 + 0.5	67.66 ± 2.51d	5.23 ± 0.45d	8.0 ± 0.57bc	4.0 ± 0.57d
3.0 + 1.5 + 0.5	62.66 ± 3.05e	5.23 ± 0.11d	7.0 ± 0.57c	4.0 ± 1.15d

*Mean values within a column having same alphabet are not significantly different (p < 0.05) according to Duncan’s multiple range test (DMRT). Data are the mean and standard deviation for n = 3 independent experiments.*

### Induction of Roots, *in vitro* Flowering, and Fruit Set

**FIGURE 4:**
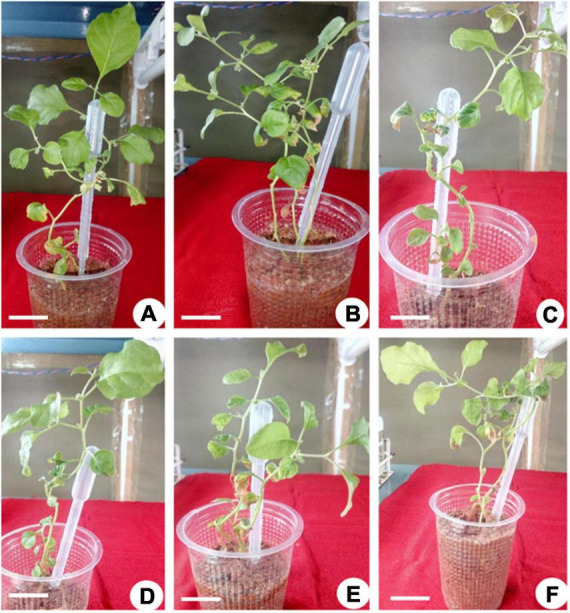
*Ex vitro* flowering in 30-day-old plantlets of *Withania somnifera*. Flower formation from **(A)** 6th node (scale bar = 13.7 cm), **(B)** 7th node (scale bar = 14.4 cm), **(C)** 11th node (scale bar = 17.5 cm), **(D)** 10th node (scale bar = 12.8 cm), **(E)** 10th node (scale bar = 12.5 cm), and **(F)** 12th node (scale bar = 17.6 cm).

The elongated shoots (4–6 cm) were transferred to a half-strength MS medium or supplemented with varying concentrations of IBA (0.5–2.0 mg L^–1^) for root induction. Data on the percentage of rooting, number of *in vitro* flowers, number of fruits, number of roots, fresh weight (FW) and dry weight (DW), and percentage of hardening were recorded (Table 4). The maximum rooting percentage (84.0%) was noted for IBA (1.5 mg L^–1^), followed by IBA (1.0 mg L^–1^), IBA (2.0 mg L^–1^), and IBA (0.5 mg L^–1^). A maximum number of flowers (18.0) was noted on IBA (1.0 mg L^–1^) during the rooting of shoots, followed by IBA (1.5 mg L^–1^), IBA (0.5 mg L^–1^), and IBA (2.0 mg L^–1^) (Figures 1C, 2D,E). The maximum number of fruits (13.0), highest root length (6.90 cm), FW (4.63 g), and DW (0.43 g) were noted on IBA (1.0 mg L^–1^), followed by IBA (1.5 mg L^–1^). The plantlet with developed roots was removed carefully from culture vessels, gently washed under tap water, transferred to a plastic cup containing sterile soil, and hardened in the tissue culture room at 25 ± 2.0°C with a photoperiod of 16 h light and 8 h dark. The maximum percentage of hardening (82.66%) was noted on IBA (1.0 mg L^–1^), followed by IBA (1.5 mg L^–1^) (Figures 1D, 2F). Furthermore, continuous *ex vitro* flowering and fruit setting were observed (Figures 4A–F). The ripe fruits were collected and shade-dried, and seeds were collected from fruits and stored at room temperature. Seeds showed a substantial germination percentage under *in vitro* and *ex vitro* conditions.

**TABLE 4:** Effect of different combinations of IBA on *in vitro* flowering and rooting responses in *Withania somnifera*.

MS + IBA (mgL^–1^)	Percentage rooting	Number of flowers	Number of fruits	Root length (cm)	Number of roots	Growth (g)		Percentage of hardening
						FW	DW	
1/2 MS	15.66 ± 6.02d	5.0 ± 0.57d	4.0 ± 1.15c	4.20 ± 0.40cd	7.0 ± 1.52e	1.52 ± 0.38de	0.19 ± 0.01d	54.33 ± 4.04d
1/2 MS + 0.5	36.66 ± 6.11c	10.0 ± 3.05c	6.0 ± 2.51bc	5.76 ± 0.45b	11.0 ± 2.08c	2.14 ± 0.15d	0.24 ± 0.03cd	67.0 ± 3.10c
1/2 MS + 1.0	56.33 ± 6.50b	18.0 ± 1.52a	13.0 ± 2.51a	6.90 ± 0.36a	17.0 ± 1.52a	4.63 ± 0.17a	0.43 ± 0.01a	82.66 ± 4.16a
1/2 MS + 1.5	84.0 ± 3.60a	14.0 ± 1.52b	8.0 ± 0.57b	5.76 ± 0.25b	15.0 ± 2.08ab	3.59 ± 0.34b	0.38 ± 0.03ab	80.33 ± 2.51ab
1/2 MS + 2.0	56.33 ± 3.21b	10.0 ± 1.52c	6.0 ± 20bc	4.76 ± 0.25c	10.0 ± 1.52cd	3.0 ± 0.11bc	0.29 ± 0.02c	79.66 ± 1.52b

*Mean values within a column having same alphabet are not significantly different (p < 0.05) according to Duncan’s multiple range test (DMRT). Data are the mean and standard deviation for n = 3 independent experiments.*

### Adventitious Root Initiation and Culture

**FIGURE 5:**
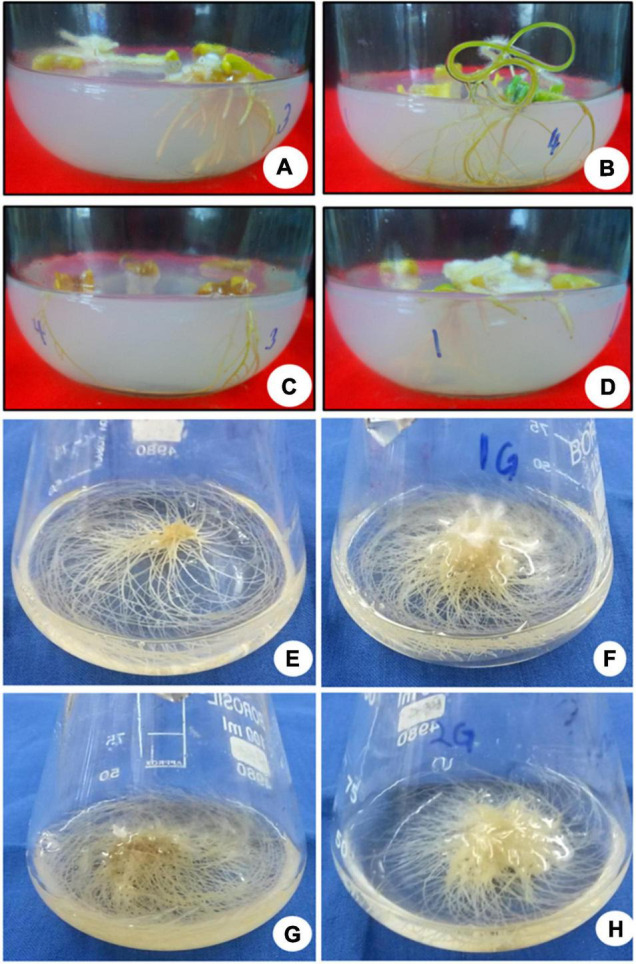
Induction and culture of adventitious roots. **(A)** Profuse growth of thick ramified roots after 25 days, **(B)** active root growth observed at 25 days, **(C)** thin, brittle roots, turned brown after 30 days, **(D)** thick roots, showing a hint of callogenesis at midrib after 40 days, **(E)** adventitious roots in MS basal media, **(F)** roots in MS + 0.5 mg L^–1^ indole-3-acetic acid (IAA) + 1.0 mg L^–1^ IBA, **(G)** MS + 0.5 mg L^–1^ IAA + 1.0 m gL^–1^ IBA + 0.20 mg L^–1^ gibberellic acid (GA_3_), and **(H)** MS media + 0.5 mg L^–1^ IAA + 2.0 mg L^–1^ IBA.

The adventitious root was induced from leaf segments (5 mm) of *in vitro* grown seedlings on MS medium supplemented with different PGRs either alone or in combination with GA_3_ (0.20 mg L^–1^) (Table 5). Among all combinations, the IAA (0.5 mg L^–1^) + IBA (1.0 mg L^–1^) + GA3 (0.2 mg L^–1^) and IAA (0.5 mg L^–1^) + IBA (2.0 mg L^–1^) + GA_3_ (0.2 mg L^–1^) exhibited a higher growth with maximum FW (2.56 g) and DW (0.81 ± 1.11), respectively, followed by IAA (0.5 mg L^–1^) + IBA (2.0 mg L^–1^) and IAA (0.5 mg L^–1^) + IBA (1.0 mg L^–1^) (Figures 5A–H).

**TABLE 5:** Responses of adventitious root formation from leaf culture.

Plant growth regulators (mgL^–1^)			Level of growth	Weight (g)	
		
IAA	IBA	GA_3_		Fresh weight	Dry weight
0.5	1	–	+	1.63 ± 0.15c	0.24 ± 0.02bc
0.5	2	–	++	1.96 ± 0.15b	0.19 ± 0.0d
0.5	1	0.20	+++	2.56 ± 0.20a	0.24 ± 0.01bc
0.5	2	0.20	+++	2.26 ± 0.15ab	0.81 ± 1.11a

*The experiment was performed in triplicate with 3 explants each. +- less growth, ++ - moderate growth, +++- higher growth. Mean values within a column having the same alphabet are not significantly different (p < 0.05) according to Duncan’s multiple range test (DMRT). Data are the mean and standard deviation for n = 3 independent experiments.*

### Expression of Withanolide Biosynthetic Pathway Genes

**FIGURE 6:**
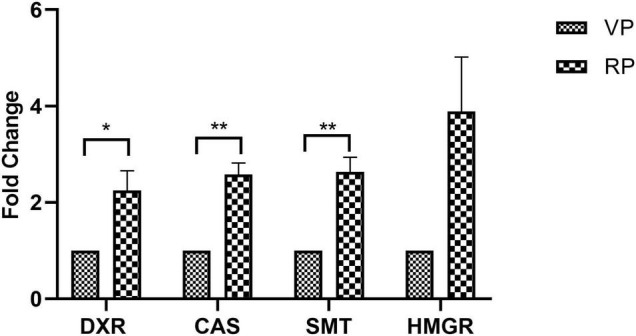
Expression of selected withanolide biosynthetic pathway genes analyzed by qRT-PCR in the leaves of *Withania somnifera* from vegetative (VP) and reproductive (RP) growth phases. The data were represented as mean ± SEM of fold change with respect to VP phase (*n* = 3) and multiple *t*-test were performed. Significant difference at *P > 0.05; **P > 0.01.

The investigation revealed an increased fold change in the expression of all genes in the reproductive phase when compared to that of genes in the vegetative phase. The maximal level of fold change expression was observed in the *HMGR* gene, followed by *SMT* and *CAS*, and a minimal level of fold change expression was observed in the *DXR* gene. Reproductive phase plants revealed a higher expression of withanolide biosynthetic pathway genes, which was confirmed by a significantly higher expression of *HMGR* (3.88-fold) followed by that of *SMT* (2.63-fold, *P* = 0.0059), *CAS* (2.58-fold, *P* = 0.0025), and *DXR* (2.24-fold, *P* = 0.0375) in comparison with their levels in the vegetative phase (Figure 6).

### Quantitative Analysis of Withanolides

**FIGURE 7:**
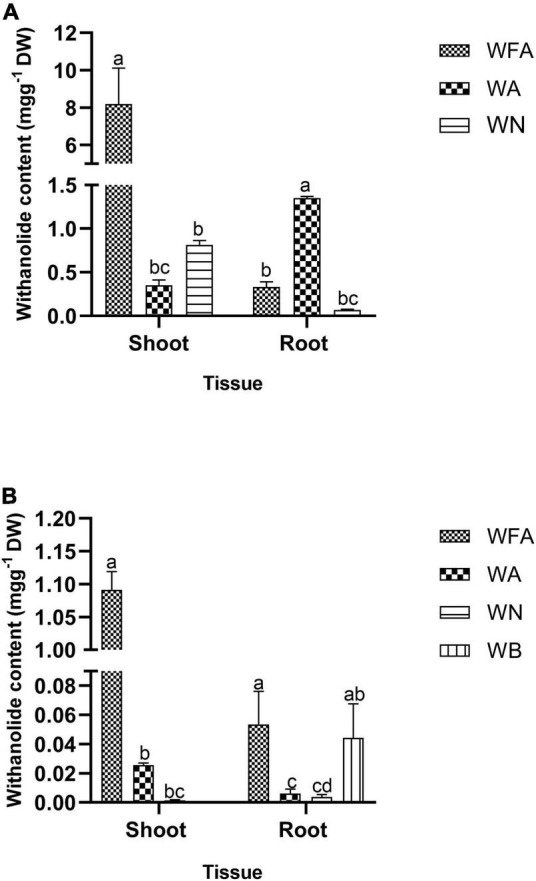
Withanolide content (mg g^–1^ DW) of *Withania somnifera*. **(A)** RP and **(B)** VP. Mean values within a column having the same alphabet are not significantly different (*p* = 0.05) according to Duncan’s multiple range test (DMRT) (*n* = 3).

**FIGURE 8:**
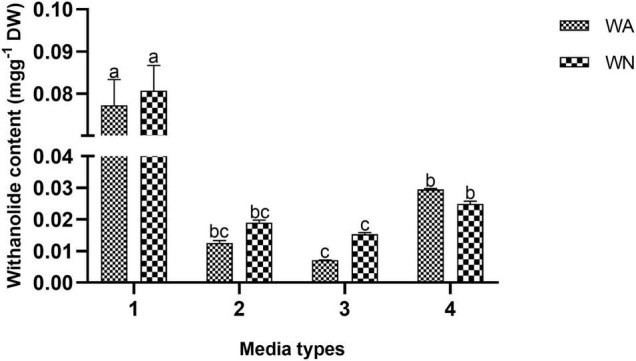
Withanolide A and withanone content (mg g^–1^ DW) from root cultures of *Withania somnifera*. (1) MS + 0.5 mg L^–1^ IAA + 1.0 mg L^–1^ IBA, (2) MS + 0.5 mg L^–1^ IAA + 2 mg L^–1^ IBA, (3) MS + 0.5 mg L^–1^ IAA + 2 mg L^–1^ IBA + 0.2 mg L^–1^ GA_3_, and (4) MS + 0.5 mg L^–1^ IAA + 1 mg L^–1^ IBA + 0.2 mg L^–1^ GA_3_. Mean values within a column having the same alphabet are not significantly different (*p* = 0.05) according to DMRT (*n* = 3).

Withanolide contents were quantified from the *ex vitro* grown plantlets (in vegetative and reproductive phases) and adventitious roots. The standard curves for WFA, WA, WB, and WN were plotted with different dilutions (0.001–1.0 mg L^–1^) and were found to be linear. The regression coefficients were recorded at 1.0, 0.9994, 1.0, and 0.9996 for WFA, WA, WB, and WN, respectively, and calculated from the calibration curve and the peak area determined by the concentration. The standards of WFA, WA, WB, and WN were eluted at the retention times of 6.43, 7.9, 11.56, and 8.38 min, respectively. The quantitative analysis of withanolide contents from the shoot and root tissues of *ex vitro* reproductive phase plantlets revealed that the maximum amount of withanolides was recorded for WFA (8.199 mg g^–1^ DW), followed by WA (0.352 mg g^–1^ DW) and WN (0.813 mg g^–1^ DW) in shoots. Furthermore, a substantially lower amount of WA (1.349 mg g^–1^ DW), WFA (0.33 mg g^–1^ DW), and WN (0.067 mg g^–1^ DW) was observed (Figure 7A). However, lower amounts of WFA (1.091 mg g^–1^ DW), WA (0.025 mg g^–1^ DW), and WN (0.0015 mg g^–1^ DW) were noted in shoots, and similarly, WFA (0.053 mg g^–1^ DW), WA (0.006 mg g^–1^ DW), WN (0.003 mg g^–1^ DW), and WB (0.044 mg g^–1^ DW) were noted in vegetative phase roots (Figure 7B). In addition, differential amounts of WA and WN contents were noted with different concentrations of IBA in the adventitious root culture. Maximum WA (0.077 mg g^–1^ DW) and WN (0.080 mg g^–1^ DW) contents were recorded with IAA (0.5 mg L^–1^) + IBA (1.0 mg L^–1^), followed by IAA (0.5 mg L^–1^) + IBA (2.0 mg L^–1^) + GA_3_ (0.2 mg L^–1^). The lowest amount of WA and WN content was recorded with IAA (0.5 mg L^–1^) + IBA (1.0 mg L^–1^) + GA_3_ (0.2 mg L^–1^) (Figure 8).

### Liquid Chromatography-Mass Spectrometry Characterization and Identification of Metabolites

**FIGURE 9:**
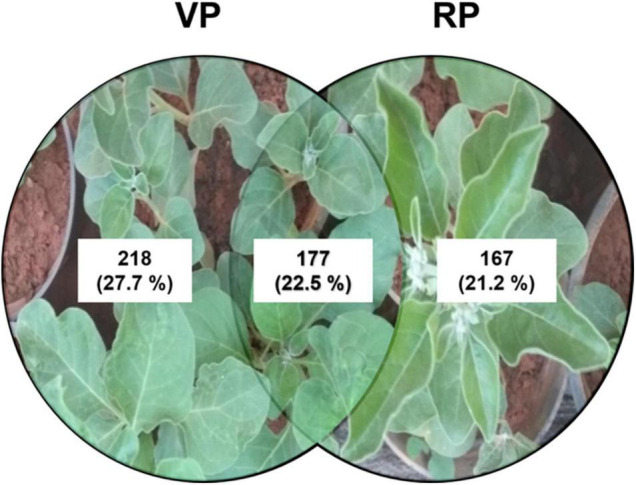
Comparative analysis and distribution of untargeted LC-MS hits of metabolites from VP and RP from whole plant extract of *Withania somnifera*. The hits were noted and analyzed for its distribution in distinct growth phases.

Metabolites from the vegetative and reproductive phases of *W. somnifera* were identified by liquid chromatography–mass spectrometry analysis. Metabolites that were common and distinct in the vegetative and reproductive phases were characterized separately after the identification. The untargeted qualitative analysis of individual metabolites was performed with a positive ionization mode. Overall, around 786 hits were observed in the vegetative and reproductive phases together. Among them, 177 were found to be common in both phases, 218 were distinctly found in the vegetative phase, and 167 were in the reproductive phase (Figure 9). Furthermore, all hits were individually searched in the plantcyc database for metabolite identification. The identified metabolites were 47 common (Supplementary Table 2), 28 distinctly vegetative (Supplementary Table 3), and 28 distinctly reproductive (Supplementary Table 4) metabolites.

## Discussion

### Multiple Shoot Initiation From Shoot Tip and Nodal Explants

Our results indicated that the effect of BAP or KIN alone or in combination coincides with previously reported shoot multiplication in *W. somnifera* in MS medium fortified with IBA and 2,4-D, using a shoot tip explant (Sen and Sharma, 1991) and the apical bud (Sivanesan, 2007). Similarly, multiple shoots were recorded with BAP and KIN using auxiliary buds (Siddique et al., 2004; Saritha and Naidu, 2007), multiple shoot regeneration was investigated with BAP, 2,4-D, and naphthalene acetic acid (NAA) using the shoot/shoot tip (Ghimire et al., 2010; Joshi and Padhya, 2010; Kanungo and Sahoo, 2011; Supe et al., 2011; Chakraborty et al., 2013), and 100% of the shooting was observed in the presence of a combination of BAP and KIN (Mir et al., 2014). In addition, multiple shoots were observed using axillary buds with BAP and NAA (Saritha and Naidu, 2007), BAP and KIN (Sabir et al., 2008), and axillary buds with BA (Fatima and Anis, 2012). The lower concentration of BAP required a longer time for breaking the bud, whereas a moderate concentration showed the maximum shoot length, number of shoots, and early bud breakage compared to the higher concentration of BAP. BAP is known for promoting cell differentiation and facilitates shoot organogenesis, whereas KIN is effective in bud breaking and rejuvenation and is known to induce a synergetic effect with other cytokinins (Shyamali and Kazumi, 2007).

Similar to shoot tip explants, nodal explants also responded differentially to various concentrations of PGRs. Our results corroborated with shoot multiplication from nodal explants of *Withania* using BA, IAA, and adenine sulfate (Sivanandhan et al., 2015), the development of multiple shoots with BAP and IAA (Sivanesan and Murugesan, 2008), and the proliferation of axillary shoots with thidiazuron (TDZ) (Fatima and Anis, 2011). Furthermore, multiple shoot formation was reported upon supplementation with BAP, NAA, and ZnSO_4_ (Fatima et al., 2011; Fatima and Anis, 2012); BAP, IAA, and spermidine (Sivanandhan et al., 2011); BAP, IAA, and L-glutamine (Sivanandhan et al., 2015), BAP and MS–B5 (Kannan and Anbazhakan, 2016), and using BAP and TDZ (Kulkarni et al., 2000).

### Multiplication and Elongation of Shoots, *in vitro* Flowering, and Adventitious Roots

This study showed a prominent percentage of elongation with BAP (2.0 mg L^–1^) and KIN (2.0 mg L^–1^) + GA_3_ (0.5 mg L^–1^), which was comparable with the results of Logesh et al. (2010). Our observations are in conformity with those of BAP, IAA, and GA_3_ combination treatment in numerous *W. somnifera* explants, such as the nodal explant (Sivanesan and Murugesan, 2008), leaf (Kumar et al., 2011), and epicotyl (Udayakumar et al., 2013). Remarkably, we have observed *in vitro* flower formation during root initiation, which is supported by the stimulatory effect of BAP and KIN with GA_3_ on *in vitro* flowering and fruiting phenomena with KIN and IAA (Saritha and Naidu, 2007), and the *in vitro* flowering with BA and IAA in *W. somnifera* (Sivanandhan et al., 2015). Flowering under *in vitro* conditions is a unique process, resulting in an addition to the other two existing reports in *W. somnifera*. This technique of rapid *in vitro* flower generation is very useful to develop seeds prior to their natural maturity; thus, it reduces the duration of the breeding cycle. *In vitro* developed seeds are important for studying genetic analysis and molecular marker technology. Furthermore, similar reports of *in vitro* flowering were described in *Swertia chirayita* (Sharma et al., 2014) with IBA, and *Andrographis lineata* (Mohammed et al., 2016) with BA and NAA.

Previously, cytokinin-induced *in vitro* flowering was reported in *Anethum graveolens* (Jana and Shekhawat, 2011), *Swertia chirayita* (Sharma et al., 2014), and *Guizotia abyssinica* (Baghel and Bansal, 2014). The impact of the synergistic effect of endogenous auxin and cytokinin facilitates *in vitro* flowering at the species level in *Micrococca mercurialis* (Jeyachandran and Bastin, 2013), *Withania* (Saritha and Naidu, 2007), *Ocimum basilicum* (Sudhakaran and Sivasankari, 2002), *Kniphofia leucocephala* (Taylor et al., 2005), and *Vitex negundo* (Thiruvengadam and Jayabalan, 2001). Hence, the addition of cytokinin hormone induces the development of *in vitro* flowering in various plants under *in vitro* conditions, similar to this study.

Our results on the rooting responses (root length, number of roots, and FW and DW) of the *in vitro* shoot with IBA showed similarity with the maximum rooting percentage in the presence of IBA (Sivanesan, 2007). Moreover, IBA also showed a positive effect in this study as well as on the *in vitro* flowering and fruiting of *Brassica campestris* (Verma and Singh, 2007), *Anthemis xylopoda* (Erdag and Emek, 2009), *Heliotropium indicum* (Bagadekar and Jayaraj, 2011), *Ceropegia pusilla* (Kalimuthu and Prabakaran, 2013), and *Brachystelma glabrum* (Lakshmi et al., 2017).

The results on adventitious root formation corroborated those of Wadegaonkar et al. (2006), who used an amalgamation of IAA and IBA for adventitious root initiation and the production of withanolides. Rangaraju et al. (2019) reported that IBA alone induced the highest adventitious root formation in different *W. somnifera* varieties. Similarly, the development of adventitious roots (using a suspension culture) and the effective accumulation of biomass were noted in *Panax notoginseng* (Gao et al., 2005) and *Echinacea purpurea* (Wu et al., 2007). Recently, an adventitious root culture was established using a B5 medium fortified with 3 mM NO_3_ and 2.5 mM phosphate, and a significant fold increase in calycosin-7-O-β-D-glucoside and antioxidant activity was recorded in *Astragalus membranaceus* (Jin et al., 2020). The development of adventitious roots from medicinal and aromatic plants has been practiced as an alternate technique for the large-scale production of bioactive compounds, especially for plants that usually reserve their medicinal compounds in their roots. In addition, for the prevention of extensive plant collection from forest areas, several rare endangered threatened (RET) species have been protected (Jiang et al., 2017).

### Expression of Withanolide Biosynthetic Pathway Genes

During stress conditions, withanolide biosynthesis is maintained through the upregulated expression of withanolide biosynthetic pathway genes, such as *CAS*, *SMT*, *DXR*, *HMGR*, and others (Singh et al., 2018), which act both as precursors and intermediates for withanolide biosynthesis. In this study, *HMGR* exhibited the highest fold change difference with respect to the vegetative phase, followed by a significant fold change difference in *SMT*, *CAS*, and *DXR* expression. Similar results were observed with the overexpression of *CAS* (3.08-fold) in the hairy root culture of *W. somnifera*, which enhanced withanolide production compared to the control groups (Saxena et al., 2017). *CAS* regulates the synthesis of cycloartenol from the precursor squalene 2,3-epoxidases, while the increased expression of *DXR* and *HMGR* promotes the production of withanolide contents in leaf tissues during drought stress conditions (Singh et al., 2014; Singh et al., 2018). *DXR* and *HMGR* are the precursors in the MEP and MVA pathways in withanolide biosynthesis, respectively. *DXR* and *HMGR* along with other genes were differentially expressed during withanolide biosynthesis when key genes were silenced through tobacco rattle virus (TRV) (Agarwal et al., 2018).

### Quantitative Analysis of Withanolides

The increased biosynthesis of WFA was recorded in the shoots trailed by WN and WA, whereas WA was higher in the roots, followed by WFA and WN in the reproductive phase than the vegetative phase plants. There were almost ten times increase in WFA, WA, and WN from the reproductive phase plants compared to vegetative phase plants. This might be due to the twofold increased expression of withanolide biosynthetic genes during the reproductive phase. Our results conform with those of Chatterjee et al. (2010), Dhar et al. (2013), and Singh et al. (2015) with a higher amount of WFA being recorded in leaves, followed by that of WN, whereas WA was higher in the roots, followed by WFA and WN. Subsequently, a higher amount of WFA was quantified *in vitro* flowers than *in vivo* fruits (Sivanandhan et al., 2015). There are several kinds of withanolides reported from different *W. somnifera* sections (Dhar et al., 2015). This study also showed a higher amount of WFA, WA, and WN in *in vitro* regenerated plants by using BAP and KIN in combination, which is similar to the results from Sabir et al. (2008). In contrast, a higher amount of WA was noted in shoots regenerated using BAP and KIN. Furthermore, a significantly higher accumulation of withanolide contents in the reproductive phase of the plants was directly proportional to the increased expression of withanolide biosynthetic pathway genes. The influence of GA_3_ on withanolide contents in adventitious roots was studied and found to be decreasing significantly than IAA/IBA treatment. Similar observations were reported by Senthil et al. (2015) where the IBA and IAA induced the highest amount of WFA and WA from the roots. In contrast, Praveen and Murthy (2010) showed the maximum amount of WA (8.8 mg g^–1^ DW) in roots with IBA supplementation (0.5 mg L^–1^).

### Liquid Chromatography-Mass Spectrometry Characterization and Identification of Metabolites

LC-MS analysis in different phases (i.e., vegetative and reproductive) identified untargeted metabolites that were later segregated as common and distinct to these two growth phases. In addition, identifying metabolites from different growth stages in *W. somnifera* can be employed to understand plant developmental processes linked to withanolide biosynthesis in leaves and roots. Common metabolites from vegetative and reproductive phases, such as L-methionine, L-tryptophan, and isatin, were identified. Metabolites, such as anthranilate, cycloglutamate, gibberellin, and others, were the characteristics of the vegetative phase. Furthermore, carnitine, amphetamine, hypusine, and other metabolites were the characteristics of the reproductive phase. Similar reports are available for *Brassica rapa* (Zou et al., 2021) and *Silybum marianum* (Khan et al., 2015) plants.

### Development of Adventitious Roots

Biomass and ginsenoside production was affected by the inoculum density of adventitious roots (Jeong et al., 2009). Here, adventitious roots were initiated in a solid medium and established using suspension culture. Our results align with the development of adventitious roots from ginseng in suspension cultures and the biosynthesis of ginsenosides (Yu et al., 2000). Similarly, adventitious root cultures were established for anthocyanin production from *Raphanus sativus* using MS medium (Betsui et al., 2004). Tropane alkaloid production was also observed in adventitious root culture development on Gamborg’s B5 medium (Min et al., 2007). Moreover, a full-strength MS medium was used to establish adventitious roots to determine biomass accumulation and production of withanolides. Similarly, Yamamoto and Kamura (1997) reported that a method for culturing adventitious roots, saikosaponin production in *Bupleurum falcatum*, and adventitious root cultures for biomass production in *Panax ginseng* were also studied (Yu et al., 2000). Furthermore, Wu et al. (2006, 2007) achieved the *in vitro* production of adventitious roots with a half-strength MS medium.

## Conclusion

Medicinal plants have recently gained major attention for their use in healthcare industries due to the trend of phytotherapeutics. Among various medicinal plants, *W. somnifera* has received increasing importance for its broad pharmacological properties against various diseases, including COVID-19. The present investigation demonstrates the influence of various PGRs on shoots and nodal explants and found that a combination of BAP and KIN enhanced shoot multiplication and growth characteristics. The obtained results showed *in vitro* flowering, increased expression of withanolide biosynthetic pathway genes and withanolide contents during the reproductive phase, and adventitious root cultures for the first time. In addition, we revealed that various metabolites present during the vegetative and reproductive phases by LC-MS analysis. These findings will improve our understanding of the withanolide biosynthetic pathway during different growth phases. In addition, *in vitro* tissue culture propagation using various PGRs provides excellent tools for propagating plants for scientific use and enhances the quantity of its commercial secondary metabolites. Our results are useful for rapid multiplication and isolation of medicinally important withanolides on industrial scales for drug research and as subsequent therapeutics in treating various chronic diseases.

## Data Availability Statement

The original contributions presented in this study are included in the article/Supplementary Material, further inquiries can be directed to the corresponding author/s.

## Author Contributions

AM, PR, and A-MB: conceptualization, funding acquisition, and supervision. SAT, PP, MR, AK, ST, and AM: performing the experiments. SAT, AK, PP, MR, KK, and AM: data analysis. AM, SAT, AK, and KK: writing the original draft. AM, SAT, AK, PR, MJ, and A-MB: review and editing with contributions of all authors. All authors contributed to the article and approved the submitted version.

## Conflict of Interest

The authors declare that the research was conducted in the absence of any commercial or financial relationships that could be construed as a potential conflict of interest.

## Publisher’s Note

All claims expressed in this article are solely those of the authors and do not necessarily represent those of their affiliated organizations, or those of the publisher, the editors and the reviewers. Any product that may be evaluated in this article, or claim that may be made by its manufacturer, is not guaranteed or endorsed by the publisher.

## Abbreviations

BAP: 6-benzylaminopurine
KIN: Kinetin
IBA: indole-3-butyric acid
IAA: indole-3-acetic acid
GA_3_: gibberellic acid
WFA: withaferin A
WA: withanolide A
WN: withanone
WB: withanolide B
WD: withanolide D
FW: fresh-weight
DW: dry-weight
PGRs: plant growth regulators.
