# Supplementary material for: Differential Gene Expression and Withanolides Biosynthesis During in vitro and ex vitro Growth of Withania somnifera (L.) Dunal
Source: Front Plant Sci. 2022 Jun 14;13:917770. doi: 10.3389/fpls.2022.917770 (PMC9237602; doi:10.3389/fpls.2022.917770)
Supplement: Supplementary file 1 [file Data_Sheet_1.docx]

**Table S1:** List of primers used for the study.

| **Sr. no.** | **Genes** | **Left primer** | **Right primer** |
| --- | --- | --- | --- |
| 1 | HMG-CoA reductase (*HMGR*) | TAGCAGGGCGTCGGAATTAC | TCTGGAGCCTGGCAAATCTG |
| 2 | 1-deoxy-D-xylulose-5-phosphate reductoisomerase (*DXR*) | GCCCGTTTGTCCTTCCACTT | CTCTGGCAAGCCTTGTATGCA |
| 3 | Cycloartenol synthase (*CAS*) | CCACTGCCGGATGGTTTATC | TAAAGAGCTCCTTTCTCAAAGACAAA |
| 4 | S-adenosyl-methionine-sterol-C-methyltransferase (*SMT*) | CAAATGACGCAGAGCAGAAA | AGGCGGAAGCTACTGAGTGA |
| 5. | *ACTIN* | GAGAGTTTTGATGTCCCTGCCATG | CAACGTCGCATTTCATGATGGAGT |

**Table S2.** Common metabolites from vegetative and reproductive phases.

| **Sr. no.** | **Proposed compounds** | **Molecular Formula** | **RT (min)** | **Ionization(ESI^+^)** | **Molecular Weight** | **Observed (*m/z*)** | **Theoretical (*m/z*)** | **Mass error (ppm, ±)** |
| --- | --- | --- | --- | --- | --- | --- | --- | --- |
| 1 | anthranil | C_7_H_5_N_1_O_1_ | 11.155 | [M + H]^+^ | 118.0295 | 119.0365 | 119.0371 | 10 |
| 2 | aminomethylpyrimidine | C_6_H_11_N_4_ | 10.089 | [M + H]^+^ | 120.064 | 138.0942 | 138.0905 | 30 |
| 3 | isatin | C_8_H_5_N_1_O_2_ | 9.291 | [M + H]^+^ | 146.0251 | 147.0326 | 147.0320 | 5 |
| 4 | 5,6-dihydroxyindole | C_8_H_7_N_1_O_2_ | 12.288 | [M + H]^+^ | 148.0411 | 149.0479 | 149.0477 | 5 |
| 5 | L-methionine | C_5_H_11_N_1_O_2_S_1_ | 12.288 | [M + H]^+^ | 148.0411 | 149.0479 | 149.0510 | 25 |
| 6 | 1-naphthaldehyde | C_11_H_8_O_1_ | 4.755 | [M + H]^+^ | 310.0924 | 156.0536 | 156.0575 | 30 |
| 7 | erythro-4-hydroxy-L-glutamate | C_5_H_8_N_1_O_5_ | 23.585 | [M + H]^+^ | 162.0362 | 163.0435 | 163.0481 | 30 |
| 8 | N-dimethylethanolamine phosphate | C_4_H_11_N_1_O_4_P_1_ | 33.107 | [M + H]^+^ | 168.0469 | 169.0542 | 169.0504 | 30 |
| 9 | N-acetyl-L-glutamate 5-semialdehyde | C_7_H_10_N_1_O4 | 4.754 | [M + H]^+^ | 172.0622 | 173.0696 | 173.0688 | 5 |
| 10 | gramine | C_11_H_15_N_2_ | 26.379 | [M + H]^+^ | 173.1079 | 174.1152 | 174.1157 | 5 |
| 11 | 4-hydroxy-4-methyl-L-glutamate | C_6_H_10_N_1_O_5_ | 29.935 | [M + H]^+^ | 176.0513 | 177.0586 | 177.0637 | 30 |
| 12 | difluoromethylornithine | C_6_H_13_N_2_O_2_F2 | 6.835 | [M + H]^+^ | 181.0789 | 182.0868 | 182.0867 | 5 |
| 13 | diisopropyl fluorophosphate | C_6_H_14_O_3_F_1_P_1_ | 14.019 | [M + H]^+^ | 183.0574 | 184.0646 | 184.0665 | 15 |
| 14 | N-hydroxy-L-dihomomethionine | C_7_H_14_N_1_O_3_S_1_ | 6.401 | [M + H]^+^ | 192.0678 | 193.0749 | 193.0773 | 15 |
| 15 | (indol-3-yl)pyruvate | C_11_H_8_N_1_O_3_ | 5.829 | [M + H]^+^ | 404.0999 | 203.0574 | 203.0582 | 5 |
| 16 | L-tryptophan | C_11_H_12_N_2_O_2_ | 20.902 | [M + H]^+^ | 203.0851 | 204.0929 | 204.0899 | 15 |
| 17 | triacsin C | C_11_H_17_N_3_O_1_ | 31.074 | [M + H]^+^ | 206.1355 | 207.1427 | 207.1372 | 30 |
| 18 | 9,10-phenanthroquinone | C_14_H_10_O_2_ | 9.392 | [M + H]^+^ | 192.0323 | 210.0661 | 210.0681 | 10 |
| 19 | o-topolin | C_12_H_13_N_5_O_1_ | 4.747 | [M + H]^+^ | 242.1026 | 243.1099 | 243.1120 | 10 |
| 20 | (7R)-trans-hinokiresinol | C_17_H_16_O_2_ | 5.897 | [M + H]^+^ | 251.1063 | 252.1136 | 252.1150 | 15 |
| 21 | pterostilbene | C_16_H_16_O_3_ | 5.886 | [M + H]^+^ | 233.1193 | 256.109 | 256.1099 | 5 |
| 22 | 1-(β-D ribofuranosyl)nicotinamide | C_11_H_15_N_2_O_5_ | 5.886 | [M + H]^+^ | 233.1193 | 256.109 | 256.1059 | 15 |
| 23 | UO2+ | O_2_U_1_ | 4.775 | [M + H]^+^ | 269.0292 | 270.0365 | 270.0406 | 20 |
| 24 | 7-methylinosine | C_11_H_15_N_4_O_5_ | 12.645 | [M + H]^+^ | 283.0992 | 284.1062 | 284.1121 | 25 |
| 25 | (1S,2R)-1-C-(indol-3-yl)glycerol 3-phosphate | C_11_H_12_N_1_O_6_P_1_ | 44.447 | [M + H]^+^ | 286.0552 | 287.0627 | 287.0559 | 30 |
| 26 | 7,2'-dihydroxy-4'-methoxy-isoflavanol carbocation intermediate 2 | C_16_H_17_O_5_ | 54.155 | [M + H]^+^ | 288.0924 | 289.0998 | 289.1076 | 30 |
| 27 | N-acetyl-α-D-galactosamine 1-phosphate | C_8_H_14_N_1_O_9_P_1_ | 4.771 | [M + H]^+^ | 300.0571 | 301.0646 | 301.0563 | 30 |
| 28 | peonidin | C_16_H_13_O_6_ | 4.771 | [M + H]^+^ | 300.0571 | 301.0646 | 301.0712 | 25 |
| 29 | delphinidin | C_15_H_9_O_7_ | 41.05 | [M + H]^+^ | 302.0483 | 303.0556 | 303.0505 | 20 |
| 30 | 2-R-hydroperoxy-linolenate | C_18_H_30_O_4_ | 23.221 | [M + H]^+^ | 309.2119 | 310.2192 | 310.2144 | 20 |
| 31 | (9Z,11E,14Z)-(13S)-hydroperoxyoctadeca-(9,11,14)-trienoate | C_18_H_29_O_4_ | 23.221 | [M + H]^+^ | 309.2119 | 310.2192 | 310.2144 | 25 |
| 32 | a bacteriochlorin | C_20_H_18_N_4_ | 52.72 | [M + H]^+^ | 313.1395 | 314.1468 | 314.1531 | 25 |
| 33 | (3-hydroxy-2-oxindol-3-yl)acetyl-L-aspartate | C_14_H_12_N_2_O7 | 9.413 | [M + H]^+^ | 321.0775 | 322.0847 | 322.0801 | 15 |
| 34 | (3-hydroxy-2-oxindol-3-yl)acetyl-L-aspartate | C_14_H_12_N_2_O_7_ | 9.413 | [M + H]^+^ | 321.0775 | 322.0847 | 322.0801 | 25 |
| 35 | 9-[6(RS)-8-diamino-5,6,7,8-tetradeoxy-β-D-ribo-octofuranosyl]-9H-purin-6-amine | C_13_H_23_N_7_O_3_ | 6.006 | [M + H]^+^ | 322.1701 | 323.1778 | 323.1706 | 25 |
| 36 | 6-(methylsulfanyl)hexyl-desulfoglucosinolate | C_14_H_27_N_1_O_6_S_2_ | 36.161 | [M + H]^+^ | 368.1202 | 369.1272 | 369.1280 | 5 |
| 37 | 6-hydroxyprotopine | C_20_H_19_N_1_O_6_ | 36.161 | [M + H]^+^ | 368.1202 | 369.1272 | 369.1212 | 25 |
| 38 | cyanidin 5-O-β-D-glucoside | C_21_H_19_O_11_ | 29.837 | [M + H]^+^ | 448.1118 | 449.1191 | 449.1084 | 25 |
| 39 | cyanidin-3-O-β-D-galactoside | C_21_H_20_O_11_ | 29.837 | [M + H]^+^ | 448.1118 | 449.1191 | 449.1084 | 25 |
| 40 | cyanidin-3-O-β-D-glucoside | C_21_H_19_O_11_ | 29.837 | [M + H]^+^ | 448.1118 | 449.1191 | 449.1084 | 25 |
| 41 | delphinidin-3-O-β-D-glucoside | C_21_H_19_O_12_ | 40.836 | [M + H]^+^ | 464.1063 | 465.1135 | 465.1033 | 25 |
| 42 | fusidate | C_31_H_47_O_6_ | 58.16 | [M + H]^+^ | 515.3408 | 516.3481 | 516.3451 | 10 |
| 43 | pelargonidin 3-O-sophoroside | C_27_H_30_O_15_ | 43.232 | [M + H]^+^ | 594.1719 | 595.1794 | 595.1663 | 25 |
| 44 | delphinidin 3-O-rutinoside | C_27_H_30_O_16_ | 29.813 | [M + H]^+^ | 610.1681 | 611.1753 | 611.1612 | 25 |
| 45 | delphinidin 3-O-rutinoside-7-O-glucoside | C_33_H_40_O_21_ | 27.953 | [M + H]^+^ | 772.223 | 773.2302 | 773.2140 | 25 |
| 46 | 1-α-linolenoyl-2-α-linolenoyl-phosphatidylcholine | C_44_H_76_N_1_O_8_P_1_ | 56.336 | [M + H]^+^ | 778.5437 | 779.5509 | 779.5465 | 10 |
| 47 | trifluoroalanine | C_3_H_3_N_1_O_2_F_3_ | 4.751 | [M + H]^+^ | 284.0221 | 143.0183 | 143.0194 | 10 |

**Table S3.** Distinct metabolites from vegetative phase.

| **Sr. no.** | **Proposed compounds** | **Molecular Formula** | **RT (min)** | **Molecular weight** | **Ionization (ESI^+^)** | **Observed (*m/z*)** | **Theoretical (*m/z*)** | **Mass error (ppm, ±)** |
| --- | --- | --- | --- | --- | --- | --- | --- | --- |
| 1 | anthranilate | C_7_H_6_N_1_O_2_ | 9.348 | 136.0405 | [M + H]^+^ | 137.0478 | 137.0476785 | 5 |
| 2 | muconolactone | C_6_H_5_O_4_ | 5.56 | 141.0229 | [M + H]^+^ | 142.0294 | 142.0266087 | 20 |
| 4 | 3-(indol-3-yl)acrylate | C_11_H_8_N_1_O_2_ | 54.042 | 186.057 | [M + H]^+^ | 187.0642 | 187.0633285 | 5 |
| 5 | cyclogutamate | C_8_H_12_N_1_O_4_ | 4.733 | 186.0742 | [M + H]^+^ | 187.0823 | 187.0844579 | 20 |
| 6 | γ-methylphosphinothricin | C_6_H_13_N_1_O_4_P_1_ | 44.045 | 194.0627 | [M + H]^+^ | 195.0699 | 195.0660445 | 20 |
| 7 | L-willardiine | C_7_H_9_N_3_O_4_ | 22.126 | 198.0564 | [M + H]^+^ | 199.0637 | 199.0593058 | 30 |
| 8 | N-amino DAP | C_7_H_14_N_3_O_4_ | 23.763 | 204.0936 | [M + H]^+^ | 205.1008 | 205.106256 | 30 |
| 9 | a jasmonate | C_12_H_17_O_3_ | 17.286 | 209.1202 | [M + H]^+^ | 210.1274 | 210.1255944 | 10 |
| 10 | 4-methoxy-3-indolylmethylisothiocyanate | C_11_H_10_N_2_O_1_S_1_ | 5.381 | 217.0392 | [M + H]^+^ | 218.0464 | 218.0513836 | 30 |
| 11 | pyridoxine 5'-phosphate | C_8_H_10_N_1_O_6_P_1_ | 5.73 | 248.0352 | [M + H]^+^ | 249.0425 | 249.0402236 | 10 |
| 12 | O-phospho-L-tyrosine | C_9_H_10_N_1_O_6_P_1_ | 6.04 | 260.0347 | [M + H]^+^ | 261.043 | 261.0402236 | 15 |
| 13 | indigo | C_16_H_10_N_2_O_2_ | 5.577 | 261.0663 | [M + H]^+^ | 262.0735 | 262.0742276 | 5 |
| 14 | hypoglycin B | C_12_H_17_N_2_O_5_ | 5.887 | 269.1162 | [M + H]^+^ | 270.1235 | 270.1215717 | 10 |
| 15 | methoxyanigorufone | C_20_H_14_O_2_ | 5.952 | 285.0939 | [M + H]^+^ | 286.1012 | 286.0993797 | 10 |
| 16 | (1S,2R)-1-C-(indol-3-yl)glycerol 3-phosphate | C_11_H_12_N_1_O_6_P_1_ | 43.269 | 286.0521 | [M + H]^+^ | 287.0593 | 287.0558737 | 15 |
| 17 | (-)-3'-methylcatechin | C_16_H_16_O_6_ | 5.679 | 303.0787 | [M + H]^+^ | 304.086 | 304.0946882 | 30 |
| 18 | sclareol | C_20_H_36_O_2_ | 4.878 | 307.2671 | [M + H]^+^ | 308.2754 | 308.2715304 | 20 |
| 19 | a porphyrin | C_20_H_14_N_4_ | 5.922 | 309.1124 | [M + H]^+^ | 310.1197 | 310.1218465 | 10 |
| 20 | (+)-5-deoxystrigol | C_19_H_22_O_5_ | 5.93 | 329.1382 | [M + H]^+^ | 330.1455 | 330.1467238 | 5 |
| 21 | gibberellin A7 | C_19_H_21_O_5_ | 5.93 | 329.1382 | [M + H]^+^ | 330.1455 | 330.1467238 | 5 |
| 22 | 5-(methylsulfanyl)pentyl-desulfoglucosinolate | C_13_H_25_N_1_O_6_S_2_ | 23.811 | 354.1006 | [M + H]^+^ | 355.1077 | 355.1123289 | 15 |
| 23 | lariciresinol | C_20_H_24_O_6_ | 5.913 | 359.1498 | [M + H]^+^ | 360.1571 | 360.1572885 | 5 |
| 24 | 7-deoxyloganin | C_17_H_26_O_9_ | 30.967 | 746.2883 | [M + H]^+^ | 374.1517 | 374.1576824 | 20 |
| 25 | O4,O5--dimethylthujaplicatin | C_22_H_26_O_7_ | 5.885 | 401.1681 | [M + H]^+^ | 402.1754 | 402.1678532 | 20 |
| 26 | glucoiberverin | C11H_20_N_1_O_9_S_3_ | 9.442 | 406.0345 | [M + H]^+^ | 407.0424 | 407.0378433 | 15 |
| 27 | sucrose-2-(2-methyl)butyryl-4-(2-methyl)butyryl-3-(4-methyl)pentanoate | C_28_H_48_O_14_ | 38.072 | 607.2936 | [M + H]^+^ | 608.3007 | 608.3044063 | 10 |
| 28 | cyanidin-3-O-rutinoside-5-O-β-D-glucoside | C_33_H_40_O_20_ | 29.79 | 756.2236 | [M + H]^+^ | 757.2309 | 757.2191188 | 20 |

**Table S4.**Distinct metabolites from reproductive phase.

| **Sr. no.** | **Proposed compounds** | **Molecular Formula** | **RT (min)** | **Ionization (ESI^+^)** | **Molecular weight** | **Observed *(m/z)*** | **Theoretical *(m/z)*** | **Mass error (ppm, ±)** |
| --- | --- | --- | --- | --- | --- | --- | --- | --- |
| 1 | carnitine | C_7_H_15_N_1_O_3_ | 14.463 | [M + H]^+^ | 162.1181 | 163.1254 | 163.1208434 | 30 |
| 2 | amphetamine | C_9_H_14_N_1_ | 20.776 | [M + H]^+^ | 134.0968 | 135.1042 | 135.1047994 | 5 |
| 3 | indole-3-carboxaldehyde | C_9_H_7_N_1_O_1_ | 20.11 | [M + H]^+^ | 144.0466 | 145.054 | 145.0527639 | 10 |
| 4 | 3-(aminomethyl)indole | C_9_H_11_N_2_ | 12.782 | [M + H]^+^ | 290.154 | 146.0843 | 146.0843983 | 5 |
| 5 | L-homomethionine | C_6_H_13_N_1_O_2_S_1_ | 6.844 | [M + H]^+^ | 162.0572 | 163.0645 | 163.0666994 | 20 |
| 6 | 2-hydroxy-2-(1H-indol-3-yl)acetonitrile | C_10_H_8_N_2_O_1_ | 14.335 | [M + H]^+^ | 171.0574 | 172.0647 | 172.0636629 | 10 |
| 7 | (E)-8-(methylsulfanyl)octanal oxime | C_9_H_19_N_1_O_1_S_1_ | 56.794 | [M + H]^+^ | 188.111 | 189.1183 | 189.1187349 | 5 |
| 8 | caffeine | C_8_H_10_N_4_O_2_ | 35.034 | [M + H]^+^ | 193.0785 | 194.0858 | 194.0803756 | 30 |
| 9 | (4S)-4-(5,5-dimethylcyclohex-1-en-1-yl)cyclohex-1-ene-1-carbaldehyde | C_15_H_22_O_1_ | 59.29 | [M + H]^+^ | 434.3078 | 218.1612 | 218.1670653 | 30 |
| 10 | N, N-dihydroxytrihomomethionine | C_8_H_16_N_1_O_4_S_1_ | 48.689 | [M + H]^+^ | 222.0742 | 223.0815 | 223.0878287 | 30 |
| 11 | 3-hydroxy-L-kynurenine | C_10_H_12_N_2_O_4_ | 14.257 | [M + H]^+^ | 223.0763 | 224.0836 | 224.0797069 | 20 |
| 12 | O(2),1,7,9-tetramethylurate | C_9_H_12_N_4_O_3_ | 18.802 | [M + H]^+^ | 223.0901 | 224.0973 | 224.0909403 | 30 |
| 13 | hypusine | C_10_H_25_N_3_O_3_ | 16.141 | [M + H]^+^ | 464.3251 | 233.1698 | 233.1739416 | 20 |
| 14 | stearidonate | C_18_H_27_O_2_ | 38.356 | [M + H]^+^ | 275.1948 | 276.2021 | 276.2089301 | 30 |
| 15 | ent-cassa-12,15-dien-2β-ol | C_20_H_32_O_1_ | 5.315 | [M + H]^+^ | 287.2425 | 288.2497 | 288.2453156 | 20 |
| 16 | N-heptylpantothenamide | C_16_H_32_N_2_O_4_ | 6.364 | [M + H]^+^ | 315.2222 | 316.2295 | 316.2362075 | 30 |
| 17 | oryzalide A | C_19_H_28_O_4_ | 31.445 | [M + H]^+^ | 319.1844 | 320.1917 | 320.1987594 | 30 |
| 18 | a jasmonoyl-L-leucine | C_18_H_28_N_1_O_4_ | 13.992 | [M + H]^+^ | 322.2112 | 323.2186 | 323.2096584 | 30 |
| 19 | pilosin | C_17_H_14_O_7_ | 8.791 | [M + H]^+^ | 329.0615 | 330.0688 | 330.0739528 | 20 |
| 20 | robustaquinone A | C_17_H_13_O_7_ | 8.791 | [M + H]^+^ | 329.0615 | 330.0688 | 330.0739528 | 20 |
| 21 | (6-hydroxy-indol-3-yl)acetyl-L-phenylalanine | C_19_H_17_N_2_O_4_ | 6.877 | [M + H]^+^ | 674.2357 | 338.1254 | 338.1266571 | 5 |
| 22 | caffeoylserotonin | C_19_H_18_N_2_O_4_ | 6.877 | [M + H]^+^ | 674.2357 | 338.1254 | 338.1266571 | 5 |
| 23 | CDTA | C_14_H_22_N_2_O_8_ | 5.913 | [M + H]^+^ | 345.1271 | 346.1344 | 346.1376157 | 10 |
| 24 | S-adenosyl-L-homocysteine | C_14_H_20_N_6_O_5_S_1_ | 19.572 | [M + H]^+^ | 383.1173 | 384.1246 | 384.1215885 | 10 |
| 25 | (-)-4'-demethyl-deoxypodophyllotoxin | C_11_H_20_O_7_ | 19.572 | [M + H]^+^ | 383.1173 | 384.1246 | 384.120903 | 10 |
| 26 | N-acetyl-S-geranylgeranyl-L-cysteine | C_25_H_40_N_1_O_3_S_1_ | 58.089 | [M + H]^+^ | 868.5426 | 435.2786 | 435.2807149 | 5 |
| 27 | red chlorophyll catabolite | C_35_H_36_N_4_O_7_ | 42.124 | [M + H]^+^ | 625.2522 | 626.2594 | 626.2740496 | 30 |
| 28 | 1-α-linolenoyl-2-oleoyl-phosphatidylcholine | C_44_H_80_N_1_O_8_P_1_ | 54.867 | [M + H]^+^ | 782.5711 | 783.5784 | 783.5778051 | 5 |
